# Impact of a direct-to-consumer information campaign on prescription patterns for overactive bladder

**DOI:** 10.1186/s12913-018-3147-1

**Published:** 2018-05-03

**Authors:** Masayoshi Zaitsu, Byung-Kwang Yoo, Jun Tomio, Fumiaki Nakamura, Satoshi Toyokawa, Yasuki Kobayashi

**Affiliations:** 1 Department of Public Health, Graduate School of Medicine, The University of Tokyo, 7-3-1 Hongo, Bunkyo-ku, Tokyo, 113-0033 Japan; 2 Department of Social and Behavioral Sciences, Harvard T.H. Chan School of Public Health, 677 Huntington Avenue, Kresge Building, 7th Floor, Boston, MA 02215 USA; 3 Department of Public Health Sciences, University of California, Davis, One Shields Avenue, Medical Sciences 1-C, Davis, CA 95616 USA

**Keywords:** Direct-to-consumer information, Disease awareness campaign, Prescription rate, Overactive bladder, Claims data, Interrupted time series analysis

## Abstract

**Background:**

Direct-to-consumer information (DTCI) campaign is a new medium to inform and empower patients in their decision-making without directly promoting specific drugs. However, little is known about the impact of DTCI campaigns, expanding rapidly in developed countries, on changes in prescription patterns. We sought to determine whether a DTCI campaign on overactive bladder increases the prescription rate for overactive bladder treatment drugs.

**Methods:**

We performed a 3-year retrospective cohort study of 1332 participants who were diagnosed overactive bladder but not prescribed treatment drugs prior to the examined DTCI campaign (exposure), using the health insurance claims dataset of the Japan Medical Data Center (November 19, 2010 to November 18, 2013). The DTCI campaign for overactive bladder included television, Internet, and print advertising (November 19, 2011 to December 22, 2011). We divided the study period into Pre-Campaign Year (2010–2011), Year 1 (2011–2012), and Year 2 (2012–2013). Each year began on November 19 and included Period 1 (weeks 1–5) through Period 10 (weeks 46–50). The main outcome was first-time prescription of the treatment drug for each patient, measured by 5-week periods. Using Period 10 in the Pre-Campaign Year as the referent period, we applied the Cox proportional hazard model for each period. Additionally, we performed the interrupted time series analysis (ITSA) for the first-time prescription rate per 5-week period.

**Results:**

Following the DTCI campaign, patients were about seven times more likely to receive a first prescription of a treatment drug during Period 4 in Year 1 (hazard ratio 7.09; 95% CI, 2.11–23.8; *p*-value<.01) compared with the reference period. Similar increases were also observed for subsequent Periods 5 and 6 in Year 1. The ITSA confirmed the DTCI campaign impact on the level of prescription rate (one-time increase in the regression-intercept) that increased by 1128.1 [per standardized 100,000 persons] (*p* < .05) during Period 4 in Year 1.

**Conclusions:**

The examined DTCI campaign appeared to increase the prescription rate among patients with overactive bladder for 15 weeks with a 15-week delay. Clinical outcomes of the patients with targeted diseases need to be monitored after DTCI campaigns by a future study.

**Electronic supplementary material:**

The online version of this article (10.1186/s12913-018-3147-1) contains supplementary material, which is available to authorized users.

## Background

Direct-to-consumer campaigns sponsored by the pharmaceutical industry, including direct-to-consumer advertising (DTCA) and direct-to-consumer information (DTCI) campaigns, have been a recent focus of prescription drug-use promotion in developed countries [1–4]. DTCA campaigns directly promote specific brand name medications, whereas DTCI campaigns are aimed at informing people about seeking healthcare services but should not directly promote specific drugs [4–7].

In the US and New Zealand, pharmaceutical companies have widely conducted DTCA campaigns, which have increased the use of prescription drugs in those countries [1, 3, 4]. Spending for DTCA campaigns has increased from USD 1.2 billion in 1998 to USD 4.5 billion in 2009 in the US [1, 4]. In many countries including Japan, where direct brand name drug promotion has been prohibited, DTCI campaigns (also known as disease awareness campaigns) have rapidly increased [5–7]. The current Japanese government regulations prohibit a DTCA campaign due to its direct promotion of a specific drug, but do not prohibit a DTCI campaign without such direct promotion of a specific drug. As a result, pharmaceutical industries are practically able to conduct DTCI campaigns in Japan. Although DTCI campaigns, including public awareness campaigns for specific diseases, are distinguished from the advertisement of specific drugs by law and permitted in many settings, some recent reports raised concerns about their potential advertising effects [6, 7]. However, little is known about how DTCI campaigns affect the patterns of prescription of treatment drugs.

In 2011, Astellas Pharma Inc. conducted a 5-week DTCI campaign for overactive bladder, a condition with complex urinary symptoms that affects quality of life in Japan [8]. Prior to the start of this DTCI campaign, a large survey conducted by the Japanese Continence Society in 2003 suggested that awareness about treatment for overactive bladder treatment was low [9]. The informational content of the DTCI campaign sponsored by Astellas was that overactive bladder could be cured with pharmaceutical treatment [8]. We investigated the potential change in the prescription patterns for overactive bladder treatment drugs as a result of the DTCI campaign in Japan.

We analyzed a large health claims data set to test our hypothesis that the 2011 DTCI campaign increased the prescription rate for the treatment drugs among patients with overactive bladder who had not previously used the treatment drugs.

## Methods

### Data source

The Japan Medical Data Center (JMDC), a for-profit company (www.jmdc.co.jp), collected health insurance claims of employees and their family members from several health insurance societies in Japan [10]. Using these data, the JMDC has created an anonymous and individually traceable database using their patented technology of anonymous linkage of individual claims. This database contains the following information collected by the JMDC for each claim: diagnosis information (unique Japanese disease name, International Classification of Diseases and Related Health Problems, 10th revision (ICD-10) code, and diagnosis date in year/month); prescribed drug information (drug name, dose, duration, prescription date in year/month/day); enrollee’s characteristics based on their health insurance (sex, birth year, dates of insurance enrollment, and disenrollment); and dates of individual-specific data collection periods. The data set used in this study was extracted from a total of over 3 million enrollees between 2005 and 2014 (comprising 1,640,000 employees and their 1,450,000 family members), including individuals who did not use any healthcare services.

The Research Ethics Committee of the Graduate School of Medicine, The University of Tokyo in Japan approved the study protocol (Protocol No. 10724-(1)).

### Study design

The study design was a cohort study of patients with overactive bladder who had not used the treatment drugs previously. The study period consisted of a 6-month run-in period (May 1, 2010 to November 18, 2010) and a subsequent 3-year analysis period (November 19, 2010 to November 18, 2013).

The inclusion criteria were as follows: patients with overactive bladder aged 20 years or older, who had been diagnosed with overactive bladder before May 1, 2010 and whose medical information was continuously collected by the JMDC throughout the study period. We excluded patients who had been prescribed a treatment drug during the 6-month run-in period (May 1, 2010 to November 18, 2010).

The definition of overactive bladder was that a patient had the unique Japanese disease name of overactive bladder, *kakatsudoboko*, with an ICD-10 code of N32.8 in their medical claims records. Treatment drugs were defined as anti-cholinergic drugs (solifenacin, imidafenacin, tolterodine, propiverine, oxybutynin, or flavoxate) or a beta-3 adrenergic receptor stimulator (mirabegron) based on the clinical guideline of the Japanese Continence Society at the study period [11]. Solifenacin and mirabegron are products of Astellas. Fesoterodine and darifenacin were not available for use in Japan during the study period.

### Advertising exposure

The advertising exposure of the DTCI campaign conducted was for 5 weeks (November 19, 2011 to December 22, 2011) throughout Japan via television, Internet, and print advertising [8]. During a 30-s television commercial that aired from November 19, 2011 to December 11, 2011, a well-known Japanese celebrity introduced the symptoms of overactive bladder. In the television commercial, a doctor also suggested, quote, “Overactive bladder could be cured by pharmaceutical treatment.” and “People with related symptoms should inform their healthcare providers.”

We assumed that the study population was equally exposed to the advertising campaign because it aired throughout Japan [8]. To the best of our knowledge, there was no other plausible factor to affect prescription patterns (such as a guideline update or another DTCI campaign/disease awareness campaign/public awareness campaign) during the follow-up period, except for the release of mirabegron to the pharmaceutical market on September 16, 2011 prior to the DTCI campaign.

### Main outcome, observation period, and covariates

The main outcome was first-time prescription of any treatment drug during the 3-year analysis period (November 19, 2010 to November 18, 2013) to capture the impact of the DTCI campaign on treatment drug prescription.

We assessed the impact of the DTCI campaign using a survival analysis. Aligned with the campaign start date on November 19, 2011, we divided the 3-year analysis period into three yearly periods: the Pre-Campaign Year (November 19, 2010 to November 18, 2011), Year 1 (November 19, 2011 to November 18, 2012), and Year 2 (November 19, 2012 to November 18, 2013). Because the campaign duration was 5 weeks, we created 10 observation periods of 5 weeks’ length each, starting on November 19 in each year: Period 1 (weeks 1–5), Period 2 (weeks 6–10), and consecutively to Period 10 (weeks 46–50). The period after week 50 was excluded in each year. The campaign was conducted during Period 1 in Year 1. Patients in Year 1 and Year 2 were exposed to the advertising campaign. Patients in each period had not been prescribed any treatment drugs for overactive bladder at the beginning of the period. The observation was right-censored in each period.

Covariates included 29 dummy variables indicating these 10 periods for 3 years (reference = Period 10 in the Pre-Campaign Year). Additional covariates were sex, age, and comorbidity levels. Age and comorbidity were time-varying variables and could be assessed only at the beginning of each year due to the limited data availability. The Charlson Comorbidity Index with ICD-10 was used for assessing comorbidity levels [12].

### Statistical analysis

First, to detect the change in prescription patterns owing to the DTCI campaign, Kaplan–Meier estimates were made for first-time prescriptions as the prescription rate during the overall 3-year analysis period.

Second, we ran a Cox proportional hazard model to investigate the magnitude and continuity of changes in prescription patterns as a result of the DTCI campaign. By comparing with Period 10 in the Pre-Campaign Year, we estimated hazard ratios (HR) and 95% confidence intervals (CI) for each of the remaining 29 periods as indicators of the prescription rate ratio, adjusted for sex, age, and comorbidity levels. The equation used for analysis was as follows:

$$ {\displaystyle \begin{array}{l}\left(\mathrm{First}\ \mathrm{time}\ \mathrm{prescription}\right)={\beta_{11}}^{\ast}\left(\mathrm{Female}\right)+{\beta_{12}}^{\ast}\left(\mathrm{Age}\right)+{\beta_{13}}^{\ast}\left(\mathrm{Comorbidity}\right)\\ {}\kern10em +{\beta_{21}}^{\ast}\left({\mathrm{P}}_1\ \mathrm{in}\ {\mathrm{Y}}_{\mathrm{pre}}\right)+\cdots +{\beta_{29}}^{\ast}\left({\mathrm{P}}_9\ \mathrm{in}\ {\mathrm{Y}}_{\mathrm{pre}}\right)\\ {}\kern10em +{\beta_{31}}^{\ast}\left({\mathrm{P}}_1\ \mathrm{in}\ {\mathrm{Y}}_1\right)+\cdots +{\beta_{40}}^{\ast}\left({\mathrm{P}}_{10}\ \mathrm{in}\ {\mathrm{Y}}_1\right)\\ {}\kern10em +{\beta_{41}}^{\ast}\left({\mathrm{P}}_1\ \mathrm{in}\ {\mathrm{Y}}_2\right)+\cdots +{\beta_{50}}^{\ast}\left({\mathrm{P}}_{10}\ \mathrm{in}\ {\mathrm{Y}}_2\right)\\ {}\kern10em +\mathrm{residual},\end{array}} $$

where Y represents Year (subscript is Pre (-Campaign Year), 1 or 2), and P for Period (subscript ranges from 1 to 10). In the equation above, Period 10 in the Pre-Campaign Year was the reference period. Baseline seasonal trends were evaluated using variables of the Pre-Campaign Year. In addition, we calculated crude prescription rate ratios per 100,000 person-days for periods with significant HRs as compared with the Pre-Campaign Year, to validate the magnitude of HRs.

To confirm the effect of DTCI campaign at the population-level, we performed interrupted time series analysis (ITSA) [13, 14] with aggregated data of the first-time prescription rate per each period (5-week) per standardized 100,000 persons (N_1_ of analyzed time periods = 30). We ran user-written STATA command of “itsa” with Prais-Winsten and Cochrane-Orcutt regression [13, 14]. We set the interrupted time point of the intervention at Period 4 in Year 1 in our primary ITSA analysis. Our primary ITSA analysis assumed a three-month potential time lag partly because an average interval of clinic visits was suggested to be 3 months among overactive bladder patients in Japan by a previous study [15] and also partly because it usually takes up to 3 months for overactive bladder patients to receive the first-time time treatment drug prescription when their urologists follow the Japan’s clinical guidelines that recommend other clinical procedures (i.e., screening of other possible underlying diseases, fluid intakes assessment, and pelvic-muscle floor training that may take up to 3 months in in a regular clinical setting in Japan) prior to the prescription [11, 16].

Additionally, to assess potential different time lag regarding the impact of the DTCI campaign, we performed sensitivity analyses of the ITSA with various interrupted time periods: from Period 1 to Period 5 in Year 1 (Period 5 corresponds to a 20-week delayed time lag) besides the primary ITSA analysis.

The equation used for the ITSA was as follows [13, 14]:

$$ {\displaystyle \begin{array}{l}\left(\mathrm{Aggregated}\ \mathrm{outcome}\right)={\beta}_0+{\beta_1}^{\ast}\left(\mathrm{time}\ \mathrm{since}\ \mathrm{the}\ \mathrm{start}\ \mathrm{of}\ \mathrm{the}\ \mathrm{study}\right)+{\beta_2}^{\ast}\left(\mathrm{post}-\mathrm{intervention}\right)+\\ {}\ {\beta_3}^{\ast}\left(\mathrm{time}\ \mathrm{since}\ \mathrm{the}\ \mathrm{start}\ \mathrm{of}\ \mathrm{the}\ \mathrm{study}\right)\times \left(\mathrm{post}-\mathrm{intervention}\right),\end{array}} $$

where

1. β_0_ is an “intercept” in a regression, representing the starting level of the outcome variable.
2. β_1_ is a “slope” in a regression and indicates trajectory of the outcome variable prior to the introduction of the intervention. If β_1_ is not statistically significant, the outcome level remains constant at β_0_ prior to the intervention. In this case, β_0_ also represents the level of the outcome variable immediately before the intervention.
3. β_2_ represents the “one-time” change in a “regression-intercept” or the level of the outcome that occurs in the period immediately after the intervention, which is hypothesized to be caused by the intervention.
4. β_3_ represents the “long-term” change, expressed as the “slope” difference between pre-intervention and post-intervention in a regression, which is also hypothesized to be caused by the intervention.

*Time since the start of the study* is a continuous variable, and *post-intervention* is a dummy variable (post-interrupted time point, 1; otherwise, 0).

For supplementary data analysis with a limited monthly aggregated data set extracted from the whole JMDC cohort during November 2010 to November 2012 (N_2_ of analyzed time periods = 25), we also checked the changes on different but relevant outcomes (a) the number of new diagnosis and (b) the number of newly diagnosed patients treated with medication.

All *p*-values were two-sided, and *p* < .05 was considered statistically significant. Data were analyzed using STATA/MP13.1 (StataCorp LP, College Station, TX).

## Results

Of 795,370 enrollees (458,198 male and 337,172 female) covered by the JMDC from May 1, 2010 to November 18, 2010, a total of 2812 (0.3%) patients with overactive bladder met the inclusion criteria. Excluding 1480 patients who were prescribed the treatment drugs during the run-in period, the final sample size for analysis was 1332 participants.

Baseline characteristics of participants are shown in Table 1. In the cohort, the mean age was 53 years, the proportion of female patients was 58%, and 70% of participants had one or more comorbidities. The distributions of age and comorbidity moderately changed during the analysis period.

**Table 1:** Demographic and clinical characteristics of participants with overactive bladder using no treatment drugs before each observation period

Characteristics	Pre-Campaign Year^a^	Year 1^a^	Year 2^a^	*p*-value^b^
Sample size, n	1332	1277	1157	
Sex, female, n (%)	768 (58)	731 (57)	666 (58)	.98
Age, mean (SD)	53 (13.1)	54 (13.2)	55 (13.5)	.008
Comorbidity, n (%)^c^				
0	416 (31)	337 (26)	271 (23)	<.001
1	370 (28)	364 (29)	326 (28)	
≥ 2	546 (41)	576 (45)	560 (48)	

^a^Pre-Campaign Year (November 19, 2010 to November 18, 2011); Year 1 (November 19, 2011 to November 18, 2012); Year 2 (November 19, 2012 to November 18, 2013). SD, standard deviation

^b^*p*-value: Analysis of variance or chi-square test

^c^Comorbidity: Charlson Comorbidity Index score

**Fig. 1:**
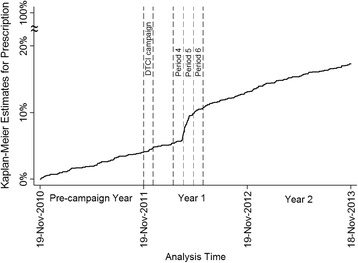
Kaplan–Meier estimate of first-time prescription for overactive bladder treatment drugs over 3-year analysis period. Period 1 started on November 19 in each year, and periods after Week 50 were included

The Kaplan–Meier estimate (Fig. 1) indicated that compared with the other periods, the prescription rate clearly increased from the end of Period 4 to Period 6 in Year 1, 10–15 weeks subsequent to the end of the DTCI campaign.

The results of the Cox proportional hazard analysis are shown in Table 2. The prescription rates from Period 4 to Period 6 in Year 1 were statistically elevated: the HRs were 7.09 (95% CI, 2.11–23.8), 13.6 (95% CI, 4.21–44.1), and 4.27 (95% CI, 1.20–15.1), respectively. These estimates are consistent with our findings (Fig. 1). After the DTCI campaign (aired during Period 1 in Year 1), patients were about seven times more likely to receive a first prescription of any treatment drug during Period 4 in Year 1 (HR = 7.09) compared with Period 10 in the Pre-Campaign Year. A weak trend in increased prescriptions was observed in Periods 1 and 8 in the Pre-Campaign Year, indicating slight seasonal changes. Age was associated with the increased first-time prescription; however, sex and comorbidity was not associated with the change (Table 2).

**Table 2:** Hazard ratios for treatment drug prescriptions adjusted for period, sex, age, and comorbidity by the cox proportional hazard model

Characteristics	Hazard Ratio (95% Confidence Interval)^a^
Age (continuous)	1.01 (1.001–1.02)*
Sex, female (versus male)	1.05 (0.80–1.37)
Charlson Comorbidity Index	
0	Reference
1	0.90 (0.62–1.29)
≥ 2	0.94 (0.68–1.31)
Observation period^b^	
Period 1 in Pre-Campaign Year (weeks 1–5)	3.22 (0.88–11.7)
Period 2 in Pre-Campaign Year (weeks 6–10)	0.97 (0.20–4.80)
Period 3 in Pre-Campaign Year (weeks 11–15)	2.27 (0.59–8.77)
Period 4 in Pre-Campaign Year (weeks 16–20)	0.98 (0.20–4.85)
Period 5 in Pre-Campaign Year (weeks 21–25)	0.98 (0.20–4.85)
Period 6 in Pre-Campaign Year (weeks 26–30)	2.29 (0.59–8.87)
Period 7 in Pre-Campaign Year (weeks 31–35)	0.99 (0.20–4.89)
Period 8 in Pre-Campaign Year (weeks 36–40)	3.31 (0.91–12.0)
Period 9 in Pre-Campaign Year (weeks 41–45)	1.00 (0.20–4.94)
Period 10 in Pre-Campaign Year (weeks 46–50)	Reference
Period 1 in Year 1 (weeks 1–5)	2.99 (0.81–11.1)
Period 2 in Year 1 (weeks 6–10)	1.34 (0.30–5.98)
Period 3 in Year 1 (weeks 11–15)	1.34 (0.30–6.00)
Period 4 in Year 1 (weeks 16–20)	7.09 (2.11–23.8)**
Period 5 in Year 1 (weeks 21–25)	13.6 (4.21–44.1)***
Period 6 in Year 1 (weeks 26–30)	4.27 (1.20–15.1)*
Period 7 in Year 1 (weeks 31–35)	3.23 (0.87–11.9)
Period 8 in Year 1 (weeks 36–40)	2.16 (0.54–8.66)
Period 9 in Year 1 (weeks 41–45)	1.45 (0.32–6.48)
Period 10 in Year 1 (weeks 46–50)	2.55 (0.66–9.86)
Period 1 in Year 2 (weeks 1–5)	2.91 (0.77–11.0)
Period 2 in Year 2 (weeks 6–10)	3.30 (0.89–12.2)
Period 3 in Year 2 (weeks 11–15)	1.84 (0.44–7.72)
Period 4 in Year 2 (weeks 16–20)	1.48 (0.33–6.62)
Period 5 in Year 2 (weeks 21–25)	1.49 (0.33–6.65)
Period 6 in Year 2 (weeks 26–30)	2.24 (0.56–8.97)
Period 7 in Year 2 (weeks 31–35)	1.50 (0.34–6.72)
Period 8 in Year 2 (weeks 36–40)	1.13 (0.23–5.60)
Period 9 in Year 2 (weeks 41–45)	1.89 (0.45–7.91)
Period 10 in Year 2 (weeks 46–50)	1.90 (0.45–7.95)

*p < .05 **p < .01 ***p < .001

^a^Sample size, *N* = 1332

^b^Pre-Campaign Year (November 19, 2010 to November 18, 2011); Year 1 (November 19, 2011 to November 18, 2012); Year 2 (November 19, 2012 to November 18, 2013). Period 1 started on November 19 in each year

The crude prescription rate ratios during Periods 4–6 in Year 1 compared with Period 10 in the Pre-Campaign Year were 7.13 (47.8 [per 100,000 person-days]/6.69 [per 100,000 person-days]), 13.7 (91.8/6.69), and 4.28 (28.7/6.69), respectively, which are similar to the magnitude of the estimated HRs during the same periods.

**Fig. 2:**
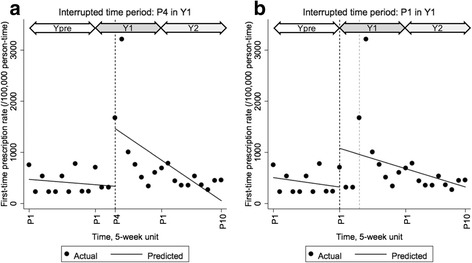
Interrupted time series analysis with different time periods for the first-time prescription rate per 5 weeks among a standardized 100,000 persons within a cohort of 1132 patients with overactive bladder who had not used the treatment drugs previously. Fitted lines were predicted with Prais-Winsten and Cochrane-Orcutt regression. The interrupted time point was set at (**a)** Period 4 in Year 1 and (**b)** Period 1 in Year 1. Y: Year, P: Period

The ITSA did not show a one-time increase in the regression-intercept during Period 1 to Period 3, as well as Period 5, in Year 1. However, as expected, the DTCI campaign significantly increased the regression-intercept at Period 4 in Year 1, which coincide with the result of our Cox model (Fig. 2 and Table 3): β_2_ = 1128.1 (95% CI, 181.7 to 2074.4; *p*-value <.01). This result suggests that the DTCI campaign raised the level of prescription rate by 2.4 times (β_2_/β_0_) with a 15-week delayed time lag. This substantial one-time increase of prescription rate was followed by the decreasing time trend (long-term effect, *p*-value <.05).

**Table 3:** Coefficients estimated with Prais-Winsten and Cochrane-Orcutt regression for the first-time prescription rate with various time periods in interrupted time series analysis model

Characteristics	Coefficient (95% Confidence Interval)^b^
Main analysis^a^	
Interrupted time period at Period 4 in Year 1	
β_0_: starting level of first-time prescription rate	471.9 (161.4 to 782.4)**
β_1_: time	− 10.4 (− 51.3 to 30.5)
β_2_: delayed effect of DTCI campaign (since Period 4 in Year 1)	1128.1 (181.7 to 2074.4)*
β_3_: time × delayed effect of DTCI campaign	−77.6 (− 175.0 to 19.7)
β_1_ + β_3_: post-intervention linear trend	− 88.1 (− 170.5 to −5.60)*
Sensitivity analyses^a^	
Interrupted time period at Period 1 in Year 1	
β_0_: starting level of first-time prescription rate	506.5 (102.0 to 911.1)*
β_1_: time	−18.4 (−94.8 to 58.0)
β_2_: effect of DTCI campaign (since Period 1 in Year 1)	757.3 (− 149.4 to 1664.0)
β_3_: time × DTCI campaign	−21.4 (− 129.1 to 86.3)
β_1_ + β_3_: post-intervention linear trend	−39.8 (−99.0 to 19.4)
Interrupted time period at Period 2 in Year 1	
β_0_: starting level of first-time prescription rate	412.7 (−8.80 to 834.2)
β_1_: time	10.7 (−62.4 to 83.8)
β_2_: effect of DTCI campaign (since Period 1 in Year 1)	552.9 (−584.2 to 1690.0)
β_3_: time × DTCI campaign	−54.4 (− 162.3 to 53.5)
β_1_ + β_3_: post-intervention linear trend	−43.7 (− 120.3 to 32.9)
Interrupted time period at Period 3 in Year 1	
β_0_: starting level of first-time prescription rate	439.3 (114.0 to 764.5)*
β_1_: time	−1.63 (−51.6 to 48.3)
β_2_: effect of DTCI campaign (since Period 1 in Year 1)	846.6 (− 234.8 to 1927.9)
β_3_: time × DTCI campaign	−62.2 (− 158.2 to 33.7)
β_1_ + β_3_: post-intervention linear trend	−63.9 (− 145.9 to 18.2)
Interrupted time period at Period 5 in Year 1	
β_0_: starting level of first-time prescription rate	393.0 (−198.7 to 984.7)
β_1_: time	12.6 (− 101.9 to 127.1)
β_2_: delayed effect of DTCI campaign (since Period 4 in Year 1)	874.4 (− 1046.5 to 2795.4)
β_3_: time × delayed effect of DTCI campaign	−106.2 (− 272.2 to 59.9)
β_1_ + β_3_: post-intervention linear trend	−93.6 (− 227.2 to 40.0)

*p < .05 **p < .01 ***p < .001

^a^Sample size, N_1_ of analyzed time periods = 30. The aggregated data samples were extracted from 1332 patients who were diagnosed with overactive bladder before May 2010 and who had not been prescribed a treatment drug during May 2010 to November 2010

^b^Coefficients and 95% CIs were estimated for the first-time prescription rate per 5 weeks among a standardized 100,000 patients with overactive bladder who had not used the treatment drugs previously

Likewise, during November 2010 to November 2012, a significant one-time increase in the regression-intercept was seen in other outcomes, i.e., the number of new diagnosis and the number of newly diagnosed patients treated with medication in the whole JMDC cohort (Additional file 1: Table S1). In addition, for the number of newly diagnosed patients treated with medication, the significant inverse time trend of the slope before the DTCI campaign changed to null (Additional file 1: Figure S1 and Table S1), suggesting that patients newly diagnosed with overactive bladder were consistently prescribed medication regardless of time trends only after the DTCI campaign. In the Cox model and ITSA, the release of mirabegron was not associated with the prescription rate (data not shown).

## Discussion

This study is the first to find the actual change in prescription patterns as the result of a DTCI campaign in a cohort, by analysis of a large health claims data set at an individual level. As hypothesized, the DTCI campaign for overactive bladder increased the prescription rate for patients with overactive bladder who had not previously used the treatment drugs. The duration of this increase was 15 weeks with a 10- to 15-week lag after the end of the DTCI campaign. The magnitude of the increase was 4–13 times that of the pre-DTCI prescription rate. The aggregated ITSA confirmed that the DTCI-campaign was associated with the one-time increase of the level of prescription rate with the magnitude of 2.4 times that of the pre-DTCI campaign period, holding a similar time lag of 15 weeks. The post-DTCI decreasing slope (long-term effect) might be partly caused by this one-time increase in the prescription rate. Additional aggregated ITSA implied the DTCI-campaign’s impacts on other outcomes such as (a) positive immediate impact on the numbers of new diagnosis and newly diagnosed patients treated with medication, and (b) the positive long-term impact on the number of newly diagnosed patients treated with medication.

Our key empirical findings regarding the timing of the DTCI campaign’s impact (i.e., with a time lag of 3 months or 3 Periods in Year 1) aligned with the expected timeline of the standard clinical practices for patients with overactive bladder in Japan (e.g., the average time to prescribe the first treatment drug being 3-month and the average visit interval being 3-month) [11, 15]. For instance, for the patients who did not visit clinics routinely but was motivated to return to a clinic by the DTCI campaign (implemented in Period 1 in Year 1), their earliest clinic visit timing was Period 1 (weeks 1–5) in Year 1 that allows the earliest timing of the first-time treatment drug prescriptions between late Period 3 and Period 4 (around weeks 14–18) in Year 1 because of the clinical guideline [11]. For the patients who visited clinics every 3 months regularly, the first visits after DTCI exposure should range between Period 1 and Period 3 (around week 13) in Year 1. Then, the second visits, or the first prescriptions after three steps recommended in the guidelines [11], should occur between the latter half of Period 3 (around week 13) and early Period 6 (around week 26) in Year 1, indicating the majority of the first prescriptions should be observed in Period 4 and Period 5 in Year 1. These estimated timelines were consistent with the larger impact of the DTCI campaign during Periods 4 and 5 in Year 1 (in terms of the hazard ratio magnitude) in our main individual-level Cox model analysis, compared to that during Period 3 and Period 6 in Year 1. Moreover, our secondary aggregated-level ITSA also support these delayed effects. Namely, the ITSA showed that the first-time prescription rate was interrupted at Period 4 in Year 1 (i.e., intercept was significantly shifted upward in a regression model [*p*-value < .05]), and that the time periods representing Periods 4 through 5 in Year 1 were the two highest outlier periods (and that representing Period 6 in Year 1 was the third highest point).

The internal validity of our estimation is discussed below. The magnitude of the increase in the current study (an approximate 140%–600% increase) was much larger than those suggested in previous studies of DTCA campaigns for antidepressant drugs (5–16% increase) or influenza vaccines (5–17% increase) [3, 17]. This gap partly stems from a very low baseline rate for our study, e.g., the crude prescription rate during Period 4 in Year 1 increased from 6.69 to 47.8 [per 100,000 person-days], which is much lower than the commonly seen baseline antidepressant drugs prescription rate (60%) and influenza vaccination rate (50%). Therefore, these very low crude prescription rates and their crude ratios imply a reasonable face validity of our HR or β estimates. Although P5 in Year 1 (during March to April) seems to be an outlier, we did not observe a significant level change at P5 in Year 1 in ITSA as shown in the Results section and Table 3. Therefore, including P5 in Year 1 in the analysis does not influence the conclusion.

In the JMDC data set, 0.3% of enrollees (mean age 53 years) had a diagnosis of overactive bladder before the DTCI campaign. In a large population-based survey in 2003, the rate of physician visits for symptoms of overactive bladder was estimated at about 0.4% when restricted to respondents in their 50s, which concurs with our findings [9]. Therefore, examination of the change in prescription patterns among patients with overactive bladder who are registered in the JMDC database is warranted.

A potential confounder in our analysis is the synergistic effect between pharmaceutical representatives (who could appeal to physicians with the “novelty” of mirabegron in the sales promotion of their company’s drugs) and the release of mirabegron before the DTCI campaign. This synergy may have overestimated the effect of the DTCI campaign on increased prescription rates. Pharmaceutical representatives have played an important role in the increase of drug prescriptions in the US [18]. In the present study’s Cox model analysis, the HR of Period 9 in the Pre-Campaign Year, which corresponds to the release period of mirabegron, was not elevated (Table 2). Likewise, the ITSA did not show a one-time increase in the level of prescription rate or a long-term change by the release of mirabegron (data not shown).

Regarding the external validity of our estimation, it should be noted that the effect of the DTCI campaign is likely to be underestimated in the current study. This is because our study population was younger than most individuals with overactive bladder in Japan [9]. Compared with older or retired patients, our study subjects were current employees (and their family) and hence were more likely to face time constraints when attending medical facilities, owing to their work schedules. Therefore, the magnitudes of the effects of the DTCI campaign among all target patients may be larger than those reported earlier in this study.

Our study had some limitations. First, the absence of the relevant dataset did not enable us to measure other factors at the individual patient level (including the launch of mirabegron and potential pharmaceutical’s in-person marketing toward physicians) that might affect the prescription pattern. However, during the period when the prescription rate was increased, there was no major change that may have affected prescriptions. Second, we could not evaluate the behavior of newly diagnosed patients because we did not have data on healthy people who did not have symptoms of overactive bladder. Third, we could not evaluate the severity of overactive bladder because such information was unavailable in our data set. In addition, the limited data availability did not allow us to analyze the DTCI impacts on other patterns including total volume of prescriptions or adherence to treatment [5–7]. Therefore, further studies are needed to evaluate the impact of the DTCI campaign by incorporating these limitations.

## Conclusions

The examined DTCI campaign appeared to change the pattern of prescriptions among patients with overactive bladder, increasing the prescription rate of treatment drugs for 15 weeks and the level of prescription rate for the post-DTCI campaign period. Future studies are expected to examine whether the increased prescription rate leads to improved health outcomes.

## Additional file

Additional file 1: **Figure S1.** and **Table S1.** Interrupted time series analysis for different outcomes. (DOCX 224 kb)

## Abbreviations

CI: Confidence interval
DTCA: Direct-to-consumer advertising
DTCI: Direct-to-consumer information
HR: Hazard ratio
ICD-10: International Classification of Diseases and Related Health Problems, 10th revision
ITSA: Interrupted time series analysis
JMDC: Japan Medical Data Center
