# Supplementary material for: Impact of a direct-to-consumer information campaign on prescription patterns for overactive bladder
Source: BMC Health Serv Res. 2018 May 3;18:325. doi: 10.1186/s12913-018-3147-1 (PMC5934904; doi:10.1186/s12913-018-3147-1)
Supplement: Supplementary file 1 — Figure S1. and Table S1. Interrupted time series analysis for different outcomes. (DOCX 224 kb) [file 12913_2018_3147_MOESM1_ESM.docx]

**Figure S1. Interrupted time series analysis for different outcomes per month per 100,000 population in the cohort of Japan Medical Data Center during November 2010 to November 2012.** Prais-Winsten and Cochrane-Orcutt regression was performed for **a** the number of new diagnosis and **b** the number of newly diagnosed patients treated with medication. The interrupted time periods were set at November 2011 and December 2011. For details of interrupted time series analysis, please refer to the manuscript's Methods section.


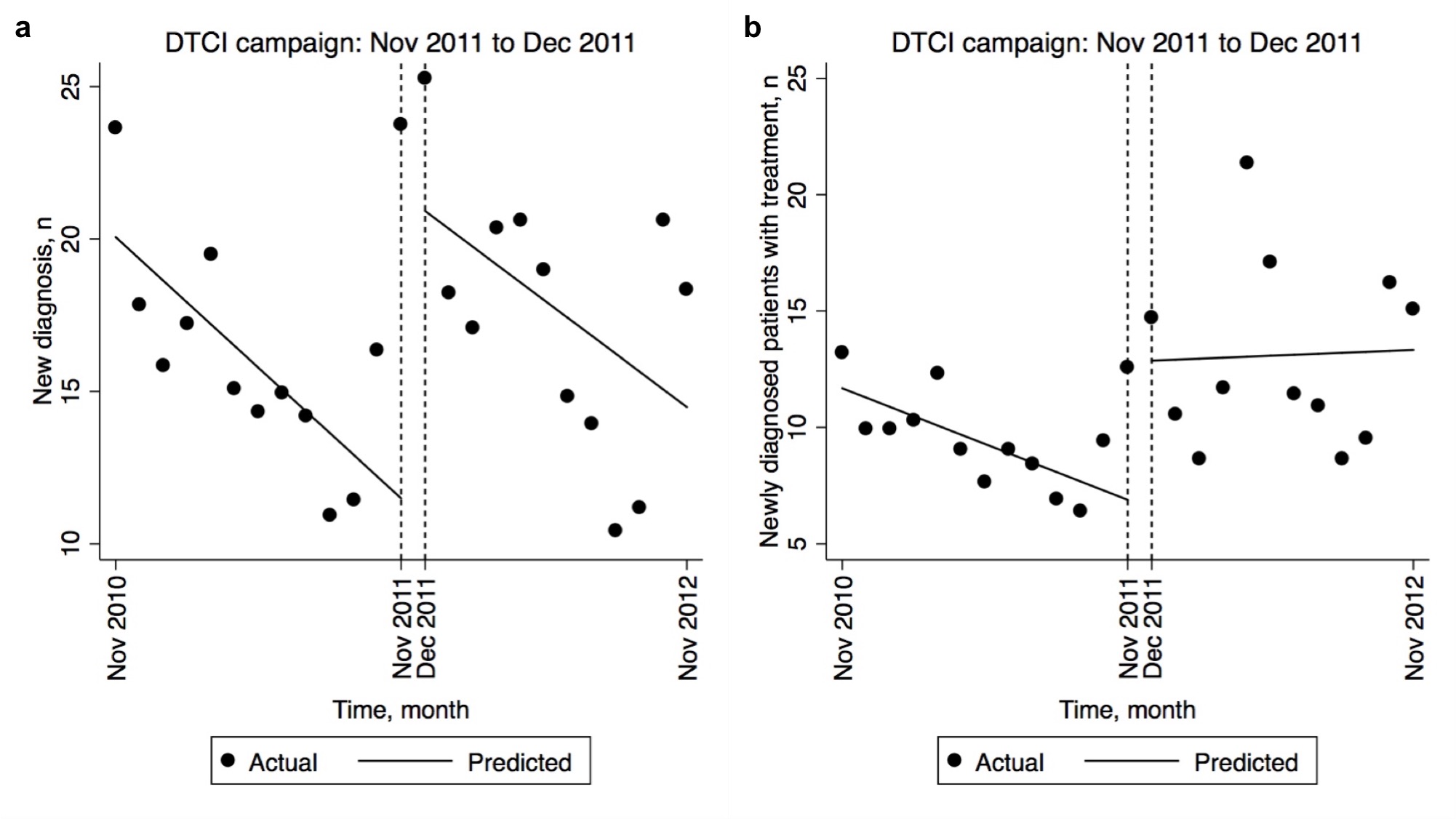


**Table S1. Interrupted time series analysis by Prais-Winsten and Cochrane-Orcutt regression for different outcomes within a cohort of 795,370 enrollees in the database of Japan Medical Data Center during November 2010 to November 2012.**

| Characteristics | Coefficient (95% CI) |
| --- | --- |
| **Monthly number for new diagnosis per 100,000 population** | |
| β_0_: starting level of new diagnosis | 20.1 (15.4 to 24.7)*** |
| β_1_: time | -0.71 (-1.46 to 0.03) |
| β_2_: November 2011 | 10.2 (5.38 to 15.0)*** |
| β_3_: time $\times$ November 2011 | 0.13 (-1.08 to 1.34) |
| β_4_: December 2011 | -0.20 (-7.04 to 6.64) |
| β_5_: time $\times$ December 2011 | *omitted* |
| β_2_ + β_4_: effect of DTCI campaign^1^ | 10.0 (2.15 to 17.9)* |
| β_1_ + β_3_ + β_5_: post-intervention linear trend | -0.58 (-1.53 to 0.36) |
|  |  |
| **Monthly number for new patients treated with medication per 100,000 population** | |
| β_0_: starting level of newly diagnosed patients treated with medication | 11.7 (9.26 to 14.1)*** |
| β_1_: time | -0.40 (-0.79 to -0.01)* |
| β_2_: November 2011 | 4.64 (1.95 to 7.34)** |
| β_3_: time $\times$ November 2011 | 0.44 (-0.32 to 1.20) |
| β_4_: December 2011 | 1.29 (-3.24 to 5.82) |
| β_5_: time $\times$ December 2011 | *omitted* |
| β_2_ + β_4_: effect of DTCI campaign^1^ | 5.93 (0.09 to 11.8)* |
| β_1_ + β_3_ + β_5_: post-intervention linear trend | 0.04 (-0.59 to 0.67) |

**p* <.05 ***p* <.01 ****p* <.001

^1^Sample size, N_2_ of analyzed time periods = 25. The aggregated data samples were extracted from 795,370 enrollees covered by the Japan Medical Data Center from November 2010 to November 2012.

^2^The effect of DTCI campaign is a combination of the effects in November and December 2011.

^3^The equation are as follows: (Aggregated outcome) =β_0_ + β_1_*(time since the start of the study) + β_2_*(post-November 2011) + β_3_*(time since the start of the study)*(post-November 2011) + β_4_*(post-December 2011) + β_5_*(time since the start of the study)*(post-December 2011); where (1) β_0_ is an “intercept” in a regression, representing the starting level of the outcome variable. (2) β_1_ is a “slope” in a regression and indicates trajectory of the outcome variable prior to the introduction of the intervention. If β_1_ is not statistically significant, the outcome level remains constant at β_0_ prior to the intervention. In this case, β_0_ also represents the level of the outcome variable immediately before the intervention. (3) β_2_ and β_4_ represent the “one-time” change in a “regression-intercept” or the level of the outcome that occurs in the period immediately after the intervention, which is hypothesized to be caused by the intervention. (4) β_3_ and β_5_ represent the “long-term” change, expressed as the “slope” difference between pre-intervention and post-intervention in a regression, which is also hypothesized to be caused by the intervention. *Time since the start of the study* is a continuous variable, and *post-November 2011* and *post-December 2011* are dummy variables (post-interrupted time point, 1; otherwise, 0).
